# Syphilis of the Endometrium

**Published:** 1888-05

**Authors:** 


### Selections.

### *SYPHILIS OF THE ENDOMETRIUM.*

Dr. T. A. Ashby, in a paper published in *The Obstetric Gazette*, says: Secondary syphilis is almost sure to manifest itself in the syphilitic woman when the disease is contracted prior to conception or during the act of conception. Ohlshausen mentions that among 657 syphilitic women, 231 miscarried, while 426

were delivered, at term, of living and dead children. Parvin states that at Lourcine 260 aborted out of 416 pregnant syphilitic women.

Abortions induced through syphilitic influence may be brought about either through maternal or fetal infection. A woman previously inoculated with syphilis will abort more readily than one who contracts the disease during pregnancy, and the danger of abortion seems to be in ratio to the period of infection. Thus contagion communicated at the time of fecundation is more likely to lead to a separation than where the poison has been introduced after the fourth month. Syphilis may be communicated through the father to the fetus, which may or may not result in its death and separation, and the mother may escape infection. On the other hand, the mothers have become infected through the fetus. A woman may have secondary syphilis and pass through the entire term of pregnancy without any manifestation of the disease in connection with the reproductive organs, but this condition of exemption may be regarded as an exception to the general rule that syphilitic women are almost sure to abort.

When pregnancy becomes established in the syphilitic subject, it invites a manifestation of the disease at the point of contact of the fetal and maternal tissues. The decidua becomes involved in a condition known as *syphiloma of the decidua*, and the placenta is so modified in its structure and mode of development as to constitute a condition recognized as placental syphilis. The extent of these changes which take place in the decidua and placenta, indicate the result of the syphilitic involvement. Separation may or may not occur, according to the extent and influence of the poison upon the maternal and fetal tissues. The placenta may be affected throughout its entire thickness, or only either the maternal or the fetal portion. When the infection occurs through the mother, the maternal portion is that which is chiefly involved, and *vice versa* when the disease approaches through the the fetus.

The local manifestation of the syphilitic virus in the decidua, as a general rule, to which I know of only two exceptions, ceases as soon as separation takes place, whether by miscarriage or labor at full term. The degenerative changes which follow the termination of gestation seem sufficient to remove the involved endometrium. This process must almost invariably take place. I have been unable to find any references in the literature of this subject to a continuation of the syphilitic manifestation upon the endometrium after labor or miscarriage. If observers have noted this condition, they have been singularly remiss in calling attention to it. That a condition of continued involvement of the mucous membrane at the placental site does occur, my experience with two cases herein related fully confirms.

The condition observed is one of continued proliferation of epithelial tissue—a highly luxuriant granulation, if I may so term it—which returns again and again after removal, resembling, in this respect, the proliferative exfoliations of an epithelial cancer. The sole origin of this condition I have referred to secondary manifestations of syphilis in the endometrium at the placental site or in this neighborhood. The disease under consideration has followed, in the two cases under my own observation, a miscarriage which was referred to the influence of the syphilitic poison. In Case II., there was a return of the endometrial trouble, after an interval of three years from date of former treatment, which could have no reference to any other causative influence. In my opinion, syphilis was the entire cause of an inflammatory condition of the endometrium, which was followed by hyperplasia of the elements of the decidua, a condition which has been described by De Sinèty as a fibroid degeneration of the villi of maternal syphilitic origin. Why the latent influence of the syphilitic virus should have manifested itself in endometrial involvement, I am unable to explain, any more than the various other singular anomalies of this disease which do not seem susceptible of rational solution. We can the more readily understand the primary outbreak of secondary syphilis in the decidua and placenta during gestation, and the continuation of the syphilitic influence upon the endometrium after miscarriage, since here we have the possible retention of placental tissue as a probable nidus for the subsequent outgrowth of granulations. In this instance, the influence of the poison is simply continued until overcome by local and constitutional treatment.

The subsequent development of syphilitic lesions upon the endometrium I can only account for upon the general assumption of a local dyscrasia in connection with the lining membrane of the uterus inviting a concentration of the specific influence upon this membrane, which resulted in hypertrophy of the glandular elements and a degenerative change in the epithelial lining of the uterine cavity.

